# Directional Association Measurement in Contingency Tables: Genomic Case

**DOI:** 10.1089/cmb.2018.0202

**Published:** 2019-03-06

**Authors:** Monika Piwowar, Tomasz KuŁaga

**Affiliations:** ^1^ Department of Bioinformatics and Telemedicine, Jagiellonian University–Medical College, Kraków, Poland.; ^2^ Faculty of Applied Mathematics, AGH University of Science and Technology, Kraków, Poland.

**Keywords:** association coefficient, associations in contingency tables

## Abstract

**Analysis of large data sets is currently a major challenge. Strong efforts are being undertaken to tackle this problem by developing new methods or modifying existing ones. The Z association method is a new method for describing directional association in contingency tables. It allows to arbitrarily group categories for each of the two variables, for which the contingency table is analyzed. The *Z* coefficient was calculated on a sample data set with gene mutations in different cancer types. Results showed some association with both gene mutations and annotation groups. Detailed results obtained for particular cancer types versus particular genes and annotation groups were in line with well-known facts in cancer genomics. The “MEUSassociation” R library allows to analyze the directional association between two categorical variables, and the mutual relationship is summarized in a contingency table, by means of the *Z* association coefficient. The method implemented in the library allows to compute the standard *Z* coefficient and to apply it in a case, where all possible singular coefficients *Z*(*A*:*B*) are computed at the same time, giving information of association between particular rows and columns. Investigating the ranked list of the highest singular coefficients allows to reduce the complexity of a large-scale data set. Both the *Z* coefficient and its R implementation are important tools in categorical data analysis.**

## 1. Introduction

Mutual relationship between two categorical phenomena can be summarized by a contingency table. There are many methods to study such relationships. They include well-known and widely used testing procedures such as the chi-square test of independence (Cochran, 1952), which has some limitations for small cell counts, and the Fisher's exact test (Fisher, 1934) or its extension the Fisher–Freeman–Halton test (Freeman and Halton, 1951) can be used for 2 × 2 or larger tables, respectively. For paired or stratified nominal data, one can use the McNemar's (McNemar, 1947) or the Cochran–Mantel–Haenszel (Cochran, 1954; Mantel and Haenszel, 1959) tests. These tests focus on testing independence between two nominal variables, possibly including additional conditions such as matching or stratifying.

Apart from statistical tests, there are also a number of measures of association calculated for contingency tables. These include the association coefficient C, the phi coefficient, or its extension the Cramer's *V* coefficient (Cramér, 1946), all being symmetric and based on the chi-squared statistics. Another example is the Goodman–Kruskal's lambda (Goodman and Kruskal, 1979) coefficient measuring the proportional reduction of error rate, which is also asymmetric where one needs to distinguish between independent and dependent variables. There is also another group of rank correlation coefficients applicable for ordinal variables, which include Spearman's rho, Goodman and Kruskal's gamma, Kendall's tau statistics, and Somers' *d* (Kendall, 1938; Somers, 1962; Goodman and Kruskal, 1979).

The *Z* coefficient described in this article belongs to the category of association coefficients. It has a purely probabilistic definition and is an asymmetric measure of association. It also coincides with Cramer's *V* coefficient in the case of *n* × 2 tables. The *Z* coefficient was successfully applied to large a data set analysis to determine connections between the structure of proteins and their biological function (Meus et al., 2006). These results appeared to be aligned with the entropy-based method (Brylinski et al., 2005). The *Z* association measurement was also used for comparative analysis of tandemly repeated trinucleotides in the human genome (Piwowar et al., 2006).

In this article, the *Z* coefficient was used to determine the association between different cancer types and different types of mutations in two ways: using a previously prepared and analyzed data set (Kandoth et al., 2013) and using an original data set with the inclusion of additional information of processes in which genes are taking part.

## 2. Methods

### 2.1. Data set

A sample data set was taken from Kandoth et al. (2013) and consisted of information related to mutated genes (with point mutations and small insertions/deletions) from 3281 tumors across 12 cancer types. Analyzes were performed on two sets:

- 12 cancer types and genes;
- 12 cancer types and annotated gene groups.

Twelve cancer types: breast adenocarcinoma (BRCA), lung adenocarcinoma (LUAD), lung squamous cell carcinoma (LUSC), uterine corpus endometrial carcinoma (UCEC), glioblastoma multiforme (GBM), head and neck squamous cell carcinoma (HNSC), colon and rectal carcinoma (COAD, READ), bladder urothelial carcinoma (BLCA), kidney renal clear cell carcinoma (KIRC), ovarian serous carcinoma (OV), and acute myeloid leukemia (LAML; conventionally called AML).

Annotated group of genes (cellular processes in which groups of mutated genes are involved): transcription factors/regulators, histone modifiers, genome integrity, receptor tyrosine kinase signaling, cell cycle, mitogen-activated protein kinase (MAPK) signaling, phosphatidylinositol-3-OH kinase (PI(3)K) signaling, Wnt/β-catenin signaling, histones, ubiquitin-mediated proteolysis, splicing, and other.

### 2.2. *Z* coefficient methodology

Given two events A and B, one could ask how much the knowledge of B helps to determine the occurrence of A. One way to measure it is to look at the ratio of error rates: one with the knowledge of B and the other without it: $( 1 - P ( A \vert B ) / \left( {1 - P \left( A \right) } \right)$. The smaller the ratio, the more information on the occurence of *A* is due to the knowledge of *B*. This idea leads to the definition of a *Z* coefficient. Given two events *A* and *B*, which define two natural partitions (family of mutually distinct events, covering the whole event space) with their respective complements $\overline A$ and $\overline B$, we define squared *Z* association coefficient between *A* and *B* by the following formula: 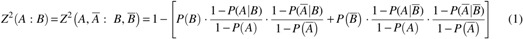


The above definition (1) can be interpreted as a value of one less than the averaged product of the family of error rate ratios calculated either with or without the knowledge of *B*. This definition can also be seen as a generalization of the Pearson correlation coefficient for two categorical variables in the following way. Having two numeric, binary variables *X* and *Y*, one can calculate the Pearson correlation coefficient. Its value is equal to the *Z* association coefficient calculated for the contingency table summarizing mutual relationship between *X* and *Y*.

Having two generic partitions *A*_1_, *A*_2_, …, *A_k_* and *B*_1_, *B*_2_, …, *B_n_*, one can define *k* × *n* contingency table $${p_{ij}}$$ with the standard notation $${p_{ij}} = P \left( {{A_i} \cap {B_j}} \right)$$, $${p_{i \cdot }}$$$$= P \left( {{A_i}} \right)$$, and $${p_{ \cdot j}} = P \left( {{B_j}} \right)$$. Now one can generalize the above definition (1) to the following: 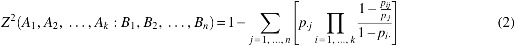


The above-defined *Z* association coefficient (2) has the following characteristics:

- ranges between 0 and 1;
- is equal to 0 in the case of independent variables (when entries in the contingency table are determined by marginal counts, or more precisely ${p_{ij}} = {p_{i \cdot }}*{p_{ \cdot j}}$);
- is equal to 1 in the case of maximal dependency, that is, where each *B_j_* determines only one possible value for some *A_i_* (in each column there is only one positive entry);
- is not symmetric (switching roles of partitions *A*_1_, *A*_2_, …, *A_k_* and *B*_1_, *B*_2_, …, *B_n_*, except the 2 × 2 tables case, generally leading to different results);
- is not monotonic (grouping particular columns or rows can both increase and decrease the value of the coefficient).

The *Z* association coefficient is also equal to Cramer's *V* coefficient (Cramér, 1946) in the case of *n* × 2 tables.

### 2.3. MEUSassociation R library

The MEUSassociation R package implements the above *Z* association coefficient. In the latest version 0.4, it provides the following functionality:

- **z_coefficient(M, col_groups = NULL, row_groups = NULL)** returns the *Z* association coefficient calculated for a given contingency table (matrix) M. It allows to specify arbitrary grouping for columns and/or rows using **col_groups** and **row_groups** parameters.
- **z_coefficient_matrix(M, col_groups = NULL, row_groups = NULL)** returns a matrix of *Z* coefficients calculated for a given contingency table (matrix) M. Each entry of a resulting matrix corresponds to the *Z* association coefficient calculated by distinguishing one particular column and row, and grouping all the remaining columns and rows into the second category. It allows to specify arbitrary grouping for columns and/or rows using **col_groups** and **row_groups** parameters. In that case, instead of calculating *Z* coefficient for each column and row, it is calculated for each column and/or row group.
- **z_coefficient_ranks(M, col_groups = NULL, row_groups = NULL)** returns ordered *Z* association coefficients calculated for each entry of a matrix M by distinguishing one particular column and row, and grouping all the remaining columns and rows into the second category. It is similar to the above **z_coefficient_matrix** function, but instead of returning results in a matrix form, it returns ordered *Z* coefficients. It allows to specify arbitrary grouping for columns and/or rows using **col_groups** and **row_groups** parameters. In that case, instead of calculating *Z* coefficient for each column and row, it is calculated for each column and/or row group.

The package also provides the following example data:

- **cancer_mutations** is a matrix (contingency table) representing different gene mutations in different cancer types [4].
- **cancer_mutations_gene_groups** is a vector of factors specifying gene groups for different gene mutations in different cancer types stored in cancer_mutations and specifies a biochemical process in which a particular gene is taking part. Information about the biochemical process in which genes are taking part can be taken from the following databases:
-> Reactome (https://reactome.org)
-> KEGG (http://www.genome.jp/kegg)

The MEUSassociation package is freely available on GitHub. The library, installation instructions, full documentation, and test data sets are available at https://github.com/mpiwowar/MEUSassociation.git. “MEUSassociation” runs under R, and does not require any additional libraries.

Executing the following short code allows one to get the results presented in the article:

- library(*MEUSassociation*)
- data(“*cancer_mutations*”)
- z_coefficient(*cancer_mutations*)
- data(“*cancer_mutations_gene_groups*”)
- z_coefficient(*cancer_mutations, row_groups = cancer_mutations_gene_groups*)
- head(z_coefficient_ranks(*cancer_mutations*))
- head(z_coefficient_ranks(*cancer_mutations, col_groups = cancer_mutations_gene_groups*))

## 3. Results

The *Z* coefficient method was used for a data set summarizing 12 different cancer types and mutated genes (Kandoth et al., 2013). The analysis was also repeated with genes grouped according to biochemical processes they are involved in. All calculations were made using the MEUSassociation R library.

The resulting *Z* association coefficient value of 0.34 suggests that there is some association between cancer types and gene mutations. When taking into account different gene annotation groups, the calculated *Z* association coefficient was equal to 0.18, suggesting that there is also some association between cancer types and biochemical processes, in which particular genes are active (Table 1). It should be noted that one should be careful when comparing *Z* coefficient values, especially for different table sizes, as the distribution of this coefficient is not well understood yet and it might tend to have higher or lower values depending on the table size.

**Table 1.** *Z* coefficient Values Calculated for Different Cancer Types and Genes, and for Different Cancer Types Versus Gene Annotation Groups

*Type of association*	Z *coefficient*
Cancer types vs. genes	0.34
Cancer types vs. gene annotation groups	0.18

**FIG. 1.**
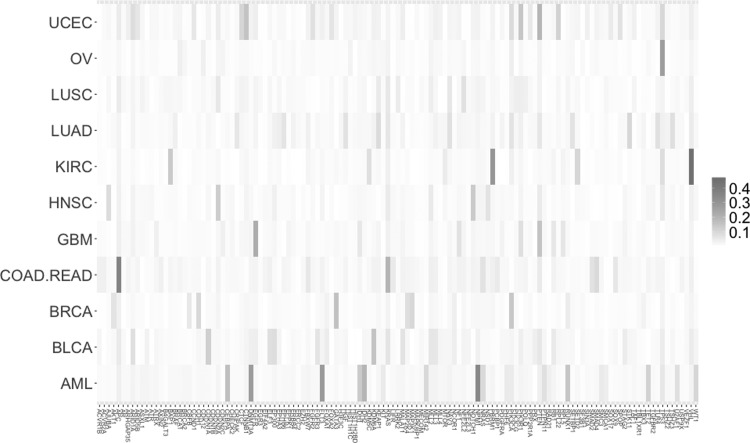
The *Z* coefficient indicating the strength of association between cancer types and genes depicted on the map with color gradation from white (minimum value) to red (maximum value).

The analysis was further extended by investigating the association between each particular cancer type and gene (Fig. 1).

The above shows that when looking at cancer types and particular genes, the strongest association exists between KIRC and von Hippel–Lindau tumor suppressor with the *Z* coefficient value of 0.47 (Table 2).

**Table 2.** Highest *Z* Coefficients Calculated for Particular Genes and Cancer Types

*Cancer type*	*Gene*	Z *coefficient*
KIRC	VHL	0.47
COAD.READ	APC	0.37
AML	NPM1	0.35
KIRC	PBRM1	0.30
AML	FLT3	0.28
AML	DNMT3A	0.27

AML, acute myeloid leukemia; COAD.READ, colon and rectal carcinoma; KIRC, kidney renal clear cell carcinoma.

**FIG. 2.**
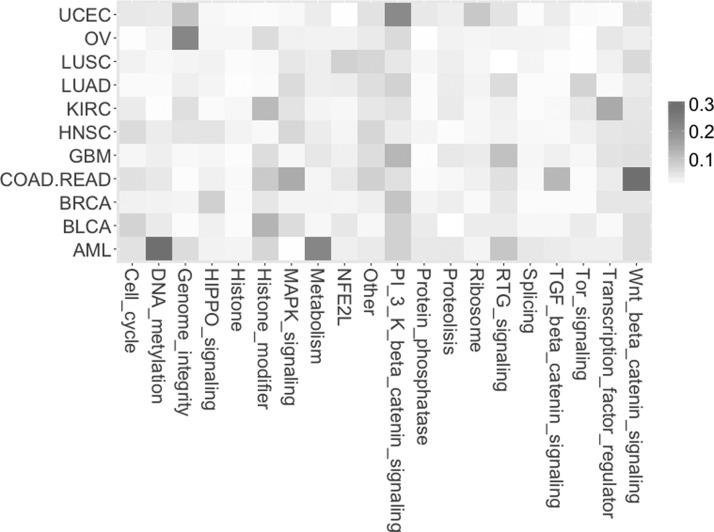
The *Z* coefficient indicating the strength of association between cancer types and gene annotation groups depicted on the map with color gradation from white (minimum value) to red (maximum value).

A similar extended analysis was done in the case of association between cancer types and gene annotation groups (Fig. 2).

The analysis shows the strongest (compared with other results) association between a combined group of the colon (COAD) and the rectal (READ) tumors (COAD.READ) and Wnt beta-catenin signaling pathway (Table 3).

**Table 3.** Highest *Z* Coefficients Calculated for Particular Gene Annotation Groups and Cancer Types

*Cancer type*	*Annotation group*	Z *coefficient*
COAD.READ	Wnt beta-catenin signaling	0.30
AML	DNA methylation	0.29
OV	Genome integrity	0.22
AML	Metabolism	0.22
UCEC	PI3K beta-catenin signaling	0.21
KIRC	Transcription factor regulator	0.15

OV, ovarian serious carcinoma; UCEC, uterine corpus endometrial carcinoma.

Literature provides strong evidence that the Wnt beta-catenin signaling pathway is very important in the READ cancer mechanism (Jung et al., 2015; Kramer et al., 2017).

## 4. Conclusion

Recent technological advances in molecular biology and other fields have given rise to numerous large-scale data sets. Analysis of such data sets imposes serious methodological challenges due to the usual large size and complex structure. The *Z* association coefficient is a tool giving valuable insight into analysis of such data sets.

“MEUSassociation” R library implements the *Z* association coefficient and allows to calculate it while grouping categories for each of two variables in an arbitrary way. In addition, the library allows for calculating the *Z* coefficient for contingency tables, evaluating the association between each particular column and row (or groups of columns and rows) while taking into account observations from the whole contingency table. These results can also be presented as a ranked list, allowing to determine row/column pairs with the highest association. It allows to reduce the complexity of high-volume data and to concentrate on the specific aspect.
